# Development of a quantitative thyroid-stimulating hormone assay system for a benchtop digital ELISA desktop analyzer

**DOI:** 10.3389/fbioe.2023.1227357

**Published:** 2023-09-21

**Authors:** Yoshiyuki Arai, Dong Wang, Miki Takeuchi, Sosuke Utsunomiya, Takuma Degawa, Atsushi Kai, Hisashi Ichikawa, Ryotaro Chiba, Toru Yoshimura

**Affiliations:** Research and Development, Abbott Japan LLC, Chiba, Japan

**Keywords:** digital ELISA, quantitative, small sample volume, TSH, ultrasensitive

## Abstract

Regular checkups for thyroid-stimulating hormone (TSH) levels are essential for the diagnosis of thyroid disease. The enzyme-linked immunosorbent assay (ELISA) technique is a standard method for detecting TSH in the serum or plasma of hospitalized patients. A recently developed next-generation ELISA, the digital immunoassay (d-IA), has facilitated detection of molecules with ultra-high-sensitivity. In this study, we developed a TSH assay system using the d-IA platform. By utilizing the ultrasensitivity of d-IA, we were able to use a sample volume of as little as 5 µL for each assay (the dead volume was 5 µL). The limits of blank, detection, and quantification (i.e., functional sensitivity), were 0.000346, 0.001953, and 0.002280 μIU/mL, respectively, and the precision of the total coefficient of variation did not exceed 10%. The correlation between serum and plasma levels indicated good agreement. Thus, our system successfully measured TSH using d-IA with a small sample volume and equal functional sensitivity to the current third generation like ARCHITECT TSH assay, which has a functional sensitivity of 0.0038 μIU/mL.

## 1 Introduction

Thyroid-stimulating hormone (TSH) is a glycoprotein produced by basophilic cells in the anterior pituitary (Pierce, 1971). TSH comprises two subunits, alpha and beta, which bind non-covalently to each other. TSH triggers production and secretion of thyroxin (T4) and triiodothyronine (T3). Synthesis and secretion of TSH are triggered by thyrotropin-releasing hormones (TRH) (Sterling and Lazarus, 1977). Because of a negative feedback system, if T3 and T4 levels increase, TSH levels decrease. The normal range of TSH varies, but typically ranges from 0.45 μIU/mL to 4.5 μIU/mL (Rugge et al., 2015), although this also depends on the age of the patient (Yoshihara et al., 2011). Under- and overproduction of thyroid hormones cause hypo- and hyperthyroidism, respectively (Golden et al., 2009; Bahn et al., 2011). Hypo- and hyperthyroidism are distinguished by the causes of dysfunction in the thyroid and pituitary glands. The causes of diseases, such as Hashimoto’s (TSH levels is 10 μIU/mL and over) and Graves (Basedow’s) diseases (TSH level below the normal reference value), are also distinguished by the levels of TSH, T3, and T4 (Chakera et al., 2012; Goichot et al., 2018). Therefore, quantitative measurement of TSH is essential for the diagnosis of thyroid disease. TSH assay systems with increasing detection sensitivity have been developed. The first generation TSH assay was developed in the 1970s. The assay was based on a radioactive immunoassay and had poor sensitivity (2.0 μIU/mL) due to its cross-reactivity with human chorionic gonadotropin (hCG). Thereafter, with use of monoclonal antibody and chemiluminescence or fluorescence probes as a conjugate, the functional sensitivity of the TSH assay had improved to 0.1 μIU/mL for the second generation and to 0.01 μIU/mL for the third generation assays (Spencer, 2023). The current third generation TSH assays enable a clear distinction between euthyroid and hyperthyroid states (Bayer, 1987; Spencer et al., 1996). For example, the functional sensitivity (the lowest analyte concentration with a 20% between-run coefficient of variation [CV] with human serum) of the ARCHITECT TSH assay (Abbott Laboratories, Chicago, IL, United States of America) is 0.0038 μIU/mL and the upper limit of detection is 100 μIU/mL. The third generation TSH tests are used in clinical laboratories with a fully automated enzyme-linked immunosorbent assay (ELISA) apparatus for high-throughput measurements.

Generally, blood samples are collected from patients on a regular basis in hospitals, and blood samples processing occurs at the hospital’s clinical laboratories or is outsourced. Commercially available *in vitro* diagnostic instruments require several tens of microliters of the sample for each assay. Therefore, multiple assays, such as thyroid-related hormone assays, require large sample volumes. For example, a sample volume of at least 267 μL, including 50 µL of dead sample volume, is required for TSH (150 µL), free T4 (45 µL), and free T3 (22 µL) measurements with Abbott laboratories’ ARCHITECT analyzer. As additional assays, such as clinical chemistry assays, may be required for diagnosis, the total sample volume will increase, which could burden patients.

Recently, single-molecule imaging-based ultrasensitive ELISA technology has been developed as the next-generation ELISA (Rissin et al., 2010; Kim et al., 2012). In this method, immunocomplexes are added to several tens of thousands of femtoliter-sized microwell reaction vessel arrays. An enzymatic reaction with the conjugate catalyzes the fluorogenic substrate in each microwell, and signals are counted “digitally.” We developed a SARS-CoV-2 antigen detection assay using a digital immunoassay (d-IA) and a benchtop-type, fully automated d-IA analyzer (Chiba et al., 2022). Taking advantage of the ultrasensitive performance of d-IA, it is possible to shorten the measurement time and to reduce the amount of specimen required while maintaining a performance comparable to that of the current third generation ELISAs. In this study, we developed a TSH assay using the benchtop d-IA platform. Our system enables the measurement of TSH in a small sample volume with equal functional sensitivity to ARCHITECT TSH assay (0.0038 µIU/mL), reducing the burden on the patient.

## 2 Materials and methods

### 2.1 Assay principle and protocol

**FIGURE 1:**
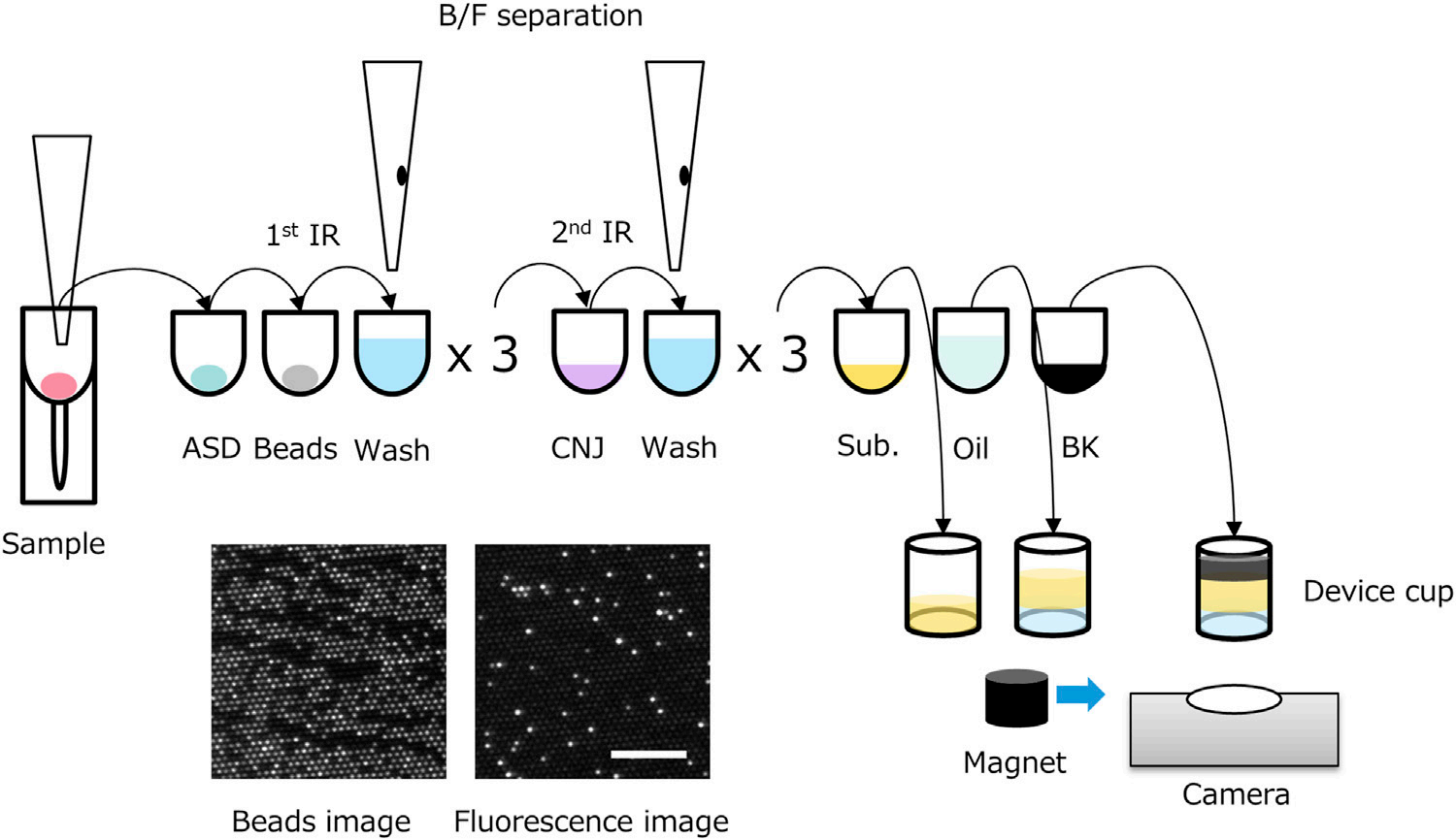
Scheme of two-step thyroid stimulating hormone (TSH) digital immunoassay (d-IA). ASD: assay specific diluent, BK: black dye, CNJ: conjugate, IR: immuno-reaction; Sub.: substrate buffer. Bottom left image: beads image, bottom right image: fluorescence image (0.5 μIU/mL of TSH). Scale bar, 100 μm.

The TSH d-IA was performed in a two-step reaction (Figure 1) on a fully automated d-IA analyzer (Chiba et al., 2022). Ten microliters of the sample was placed in a sample tube (SARSTEDT AG&Co. KG, Nümbrecht, Germany). Then, 5 µL of the sample was aspirated using an automatic pipettor (leaving a dead volume of 5 µL) and mixed with 50 µL of assay specific diluent (ASD). The sample/ASD mixture was then mixed with 50 µL of assay beads (800,000 beads in total) constructed using Magnosphere™ MS300/Tosyl beads (JSR Corporation, Tokyo, Japan) on which β-subunit monoclonal antibody against TSH (Abbott Laboratories, IL, United States of America) had been immobilized. After incubation at 37°C for 3 min, bind/free (B/F) separation was performed using Magtration™ technology (B/F separation within a disposable pipette; Precision System Science Co., Ltd., Chiba, Japan). The beads were then washed with 200 µL of ARCHITECT wash buffer (Abbott Laboratories) three times. The beads were then mixed with 50 µL of conjugate diluent with the TSH-α-subunit antibody (Abbott Laboratories) conjugated with calf intestinal alkaline phosphatase (BBI solutions, Caerphilly, United Kingdom) using trans-cyclooctene and tetrazine click chemistry (Click Chemistry Tools, Scottsdale, AZ, United States of America). Following incubation at 37°C for 2 min, B/F separation was performed, and the beads were washed with wash buffer. The beads were then suspended in 75 µL of substrate buffer containing 1 mM pyranine phosphate (Chiba et al., 2022), which was custom-made by Fujifilm Wako Pure Chemical Corporation (Osaka, Japan), and placed in a microwell cup device using a sliding magnet. The purity of pyranine phosphate was 98.5% that was measured by the ratio of absorption at 395 nm (absorption of pyranine phosphate) and 455 nm (absorption of pyranine). Oil (FC-40; 3M, St Paul, MN, United States of America) was added to the cup device (Sumitomo Bakelite Co., Ltd., Tokyo, Japan) to form femtoliter chambers. Because the FC-40 oil has a heavier specific gravity than that of the aqueous solution, the sealing oil sinks down, pushing the aqueous solution upward. Finally, 100 µL of phosphate-buffered saline containing 50 mM nigrosine (Fujifilm Wako Pure Chemical Corporation, Osaka, Japan) was added to the top of the solvent to prevent the effect of ambient light. Beads and fluorescence images were captured from the bottom of the cup device during enzymatic reactions.

### 2.2 D-IA apparatus

The d-IA was performed using a previously reported desktop analyzer (Chiba et al., 2022). Briefly, the d-IA desktop analyzer can process up to eight samples simultaneously using the same assay protocols, following the assay protocol. The d-IA desktop analyzer operates automatically after setting up the samples, reagent cartridges, pipette tips, and microwell array cup devices. The volume of each microwell was approximately 50 fL and 117,000 microwell arrays (approximately 3 mm in diameter in the field of view) were observed using a custom-made camera and illumination system. The scattering of bead images and the fluorescence of pyranine were observed under green (528 nm, MLEGRN-A1-0000-000001, CREE, Durhan, NC, United States of America) and blue LED (457 nm, XQEROY-H0-0000-000000N01, CREE) light illumination, respectively.

### 2.3 Analysis

The analysis was performed using custom Python software. The RGB color image was reconstructed from raw camera images. Ten beads and their fluorescence images were recorded. The green channel was extracted from the beads and fluorescence images. Time-lapse images were aligned with cropped bead images using the PyStackReg package (Thevenaz et al., 1998). The registered bead images were averaged, and the background was subtracted and blurred using a Gaussian kernel. Beads were detected using a local maxima algorithm under a global threshold. The fluorescence images were registered using the registration parameters of the bead images. Linear regression was performed for each pixel of the fluorescence image sequence. Consequently, slope and intercept images were reconstructed, with the former reflecting the enzyme activity. For the slope image, bright spots were detected using local maxima with a global threshold. Positive bright spots were observed on the beads.

### 2.4 Calculation of signal%

The ratio of positive bright spots in the beads (referred to as the signal%) was calculated by dividing the number of bright spots detected in the slope image by the number of beads detected (Chiba et al., 2022).

### 2.5 Analog analysis

For d-IA, the detected bright spots were digitally counted as 0 or 1. However, when the specimen concentration was high, multiple conjugates were bound to one bead. In such a case, the “true” signal of the high-concentration sample would be masked if only a digital reading was used. Therefore, in the case of a high-concentration sample, the number of conjugate bonds per bead should be estimated; that is, the average number of enzymes per bead (AEB) should be calculated, as previously described (Rissin et al., 2011). If the number of enzymes per bead is one or less (i.e., digital reading), AEB_Digital_ can be obtained using the following formula:

$$AEB_{digital}=-\ln\left(1-f_{on}\right)$$

where *f*_*on*_ is the fraction of beads with enzymes and is equivalent to the signal%. In the analog region, the fluorescence of the positive bright spots was standardized using an index based on one enzyme. This value was calculated using the following formula (Rissin et al., 2011):

$$AEB_{analog}=\frac{f_{on}\cdot \bar{I_{beads}}}{\bar{I_{single}}}$$

where $\bar{I_{single}}$ and $\bar{I_{beads}}$ were the initial velocities of mean enzyme activity in the digital and analog regions, respectively. In this study, the initial enzyme activity rate was used as an index. The time trajectories of the fluorescence intensity time series were fitted using the following formula:

$$f\left(t\right)=\left(A-offset\right)\cdot \left(1-\exp\left(-k\cdot t\right)\right)+offset$$

where *A* is the fluorescence intensity at the plateau point, *k* is the rate constant, and the offset is the value of the initial intensity estimated from the background at the initial frame. The initial velocity can be determined from the slope of the tangent at t = 0, which is calculated as the limit f(t) of t, as follows:

$${\left.\frac{df\left(t\right)}{dt}\right|}_{t=0}=\left(A-offset\right)\cdot k$$

### 2.6 Calibration

Panels with 0, 0.5, 2, 10, 40, and 100 µIU/mL of TSH were prepared (Cat. 4610-TH-010, R&D systems, Inc., MN, United States). Point-to-point calibration was used to estimate the read value using the AEB.

### 2.7 The limit of blank, limit of detection, and limit of quantification measurement

The limit of blank (LoB), limit of detection (LoD), and limit of quantification (LoQ) were measured following Clinical and Laboratory Standards Institute guideline (CLSI) EP-17-A2 (CLSI, 2012). Low-concentration panels, 0 (−0.000195), 0.0025 (0.002280), 0.005 (0.004488), 0.0075 (0.007430), 0.01 (0.009817), and 0.5 (0.499796) µIU/mL, were measured (the values in parenthesis were determined using the ARCHITECT TSH assay). The LoB was estimated using the following equation: $LoB=M_{B}+c_{p}\cdot SD_{B}$ , where *M*_*B*_ is the mean value of the 0 μIU/mL panel, *SD*_*B*_ is the estimated standard deviation of the 0 μIU/mL panel, and *B* is the number of samples. *c*_*p*_ is a multiplier that provides the 95th percentile of a normal distribution as follows: $C_{p}=1.645/\left(1-\left(1/\left(4\left(B-K\right)\right.\right)\right)$ , where *K* is the number of panels (i.e., one). Formulation of LoD calculation is described in CLSI EP-17-A2 as follows: $LoD=LoB+C_{p}\cdot SD_{L}$ where *SD*_*L*_ is the mean standard deviation of low-concentration panels, *L* is the total number of all samples, and *c*_*p*_ is a multiplier that gives the 95th percentile of a normal distribution, as follows: $C_{p}=1.645/\left(1-\left(1/\left(4\left(L-J\right)\right.\right)\right)$ where *J* is the number of panels. In this study, used *Cp* as 1.645. (Armbruster and Pry, 2008). LoQ is the concentration at which the CV% is 20%, which is the definition of the functional sensitivity for the TSH assay (Spencer et al., 1996).

### 2.8 Precision study

To evaluate the precision of the TSH d-IA, we performed a precision study using two panels (0.1 and 40 µIU/mL) and two serum samples (0.57 and 14.2 µIU/mL). The assay was performed by measuring three replicates per run, with two runs per day, for 5 days. Data were analyzed with R software using the VCA package (R Core Team, 2022; Schuetzenmeister and Dufey, 2022).

### 2.9 Correlation study

The correlation between the ARCHITECT-derived TSH levels and d-IA-derived TSH levels was evaluated in 70 samples, which included 10 patients with hyperthyroidism (Precision for Medicine), 40 normal healthy samples (Precision for Medicine), and 20 hypothyroidism samples (Complex Antibodies Inc.). The matrix difference between serum and plasma on the d-IA platform was also evaluated using 20 samples (Precision for Medicine). To estimate the slope and intercept, the Passing–Bablok method (Passing and Bablok, 1983) was used in the mcr package of R (Schuetzenmeister, 2021).

## 3 Results

### 3.1 Digital ELISA measurement

**FIGURE 2:**
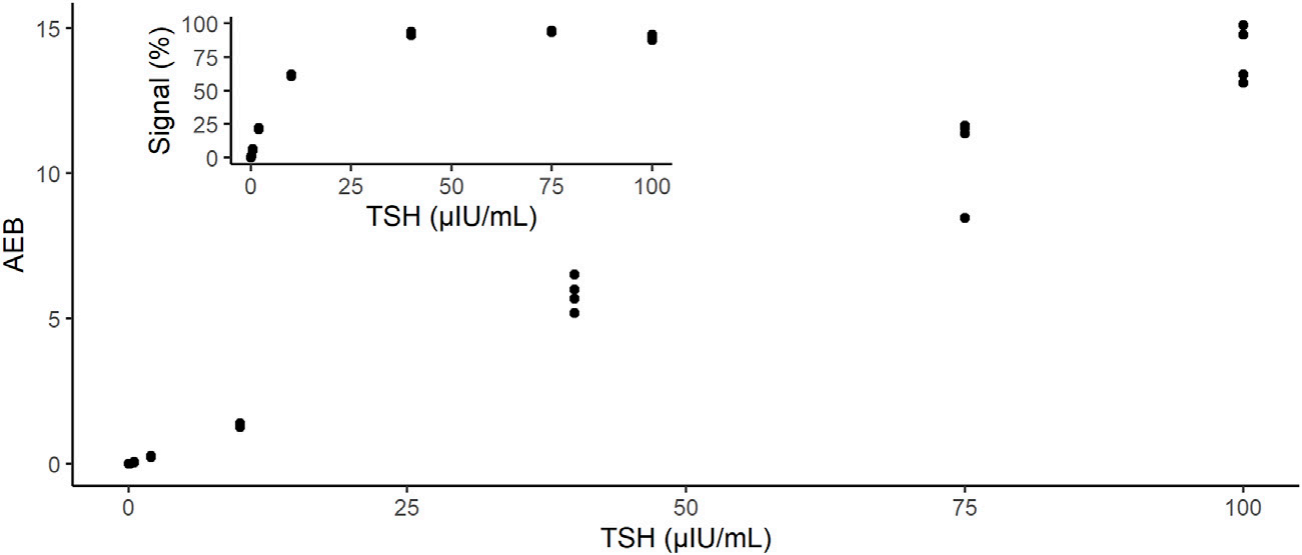
Dose-response of instrument output (AEB) against the TSH concentrations: 0, 0.5, 2, 10, 40, and 100 µIU/mL. Inset shows the output of the instrument as signal%. AEB: average number of enzymes per bead; TSH: thyroid-stimulating hormone.

To obtain calibration data, we measured recombinant TSH panels in concentrations ranging from 0 to 100 μIU/mL (Figure 2). As shown in the inset of Figure 2, the signal percentages at 10, 40, and 100 μIU/mL were saturated because an excess amount of analytes was bound to the beads. In contrast, the AEB increased with increasing TSH concentrations, up to 100 μIU/mL (Figure 2). Thus, the d-IA TSH assay enabled the measurement of at least 100 μIU/mL without sample dilution. The background fluorescence signal was markedly reduced by the addition of nigrosine (Supplementary Figure S1). We confirmed absence of cross-contamination after sealing the microwells with FC-40 oil during the observation period (Supplementary Figure S2).

### 3.2 LoB, LoD, and LoQ

**FIGURE 3:**
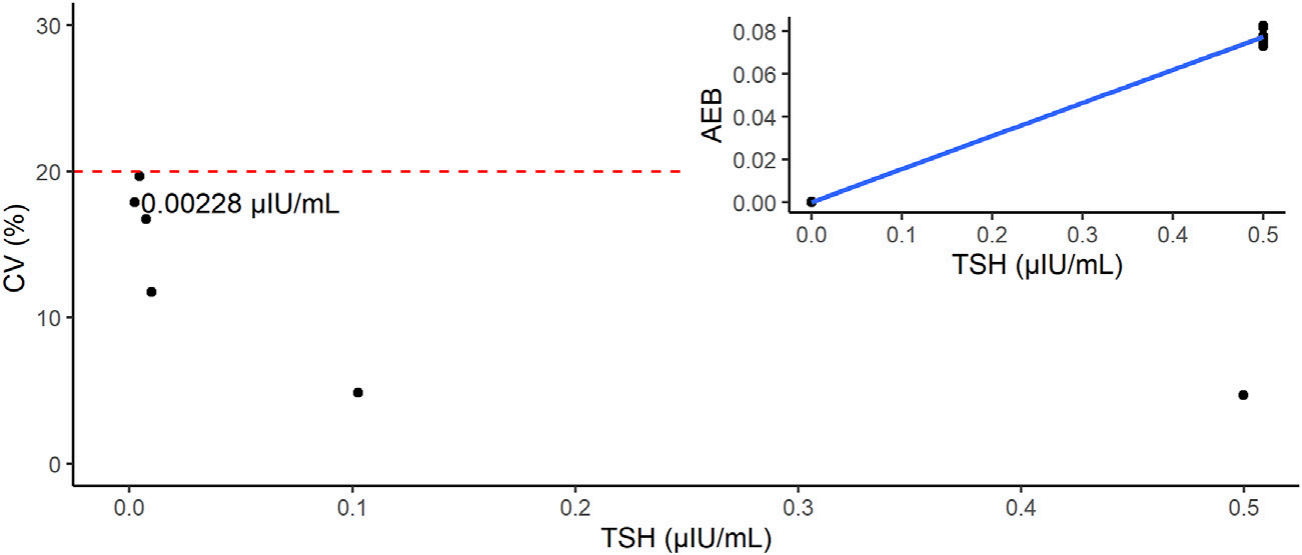
Estimation of limit of quantification (LoQ). The red dashed line indicates the line where CV = 20%. Inset shows the calibration curve of this study (point-to-point method).

We estimated the LoB, LoD, and LoQ (i.e., functional sensitivity) by measuring the low-concentration panels of the TSH samples (Table 1). The LoB and LoD values were estimated as 0.000346 and 0.001953 μIU/mL, respectively. The LoQ was estimated by CV values. Because the CV was 166.5% at 0 (−0.000195) and 17.9% at 0.0025 (0.002280) μIU/mL, the LoQ was estimated as 0.002280 µIU/mL (Figure 3).

**TABLE 1:** Summary table of the results of low-concentration TSH.

Panel (µIU/mL)	Number of samples	Mean value (µIU/mL)	Standard deviation (µIU/mL)	%CV
0	19	−0.000195	0.00032	166.5
0.0025	10	0.002280	0.00041	17.9
0.005	12	0.004488	0.00088	19.7
0.0075	15	0.007430	0.00124	16.7
0.01	15	0.009817	0.00115	11.7
0.5	7	0.499796	0.02350	4.7

TSH, thyroid-stimulating hormone; CV, coefficient of variation.

### 3.3 Precision study

**FIGURE 4:**
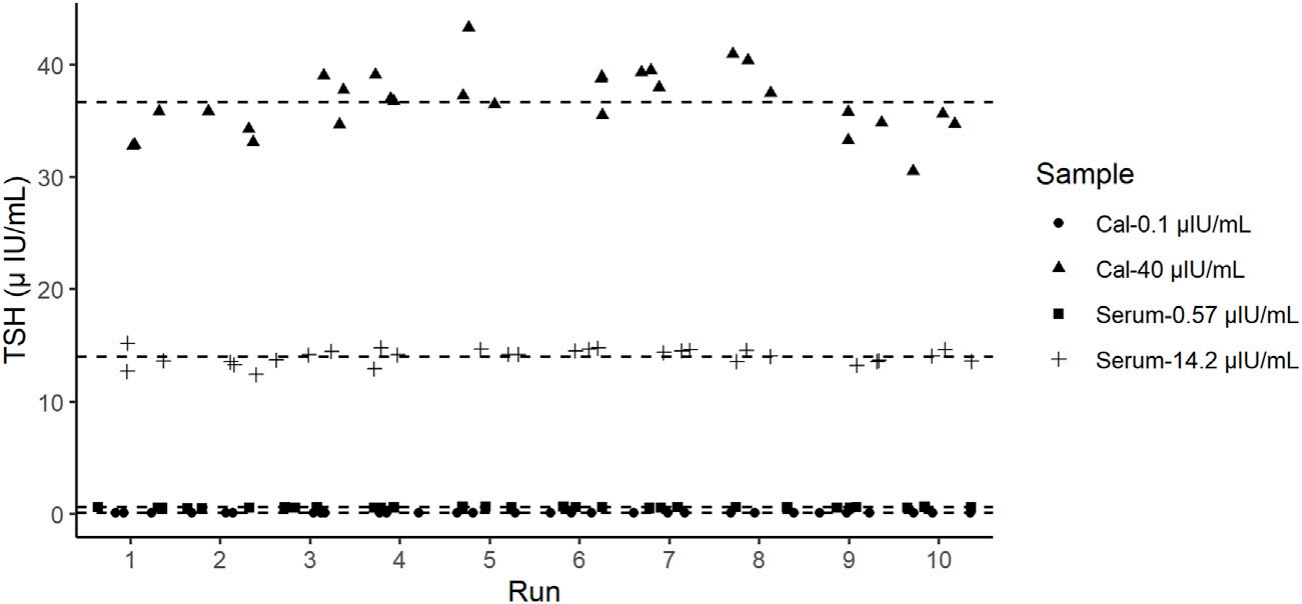
Precision test results of thyroid-stimulating hormone (TSH). Dashed lines represent the mean value of each sample.

The precision of the TSH d-IA was determined according to the CLSI EP05 (CLSI, 2014). Two controls panel (0.1 µIU/mL and 40 µIU/mL) and two serum samples (0.57 µIU/mL and 14.2 µIU/mL) were used (Figure 4). CVs among samples did not exceed 10% (Table 2).

**TABLE 2:** %CV of TSH in precision study.

Sample	Mean TSH µIU/mL	Within-run %CV	Between-run %CV	Between-day %CV	Total %CV
Control 1	0.105	6.8	2.8	4.0	8.4
Control 2	36.67	5.6	n.d.*	6.5	8.6
Serum 1	0.580	4.8	3.2	5.4	7.9
Serum 2	14.03	4.3	0.48	2.3	4.9

TSH, thyroid-stimulating hormone; CV, coefficient of variation.

### 3.4 Correlation between ARCHITECT and d-IA

**FIGURE 5:**
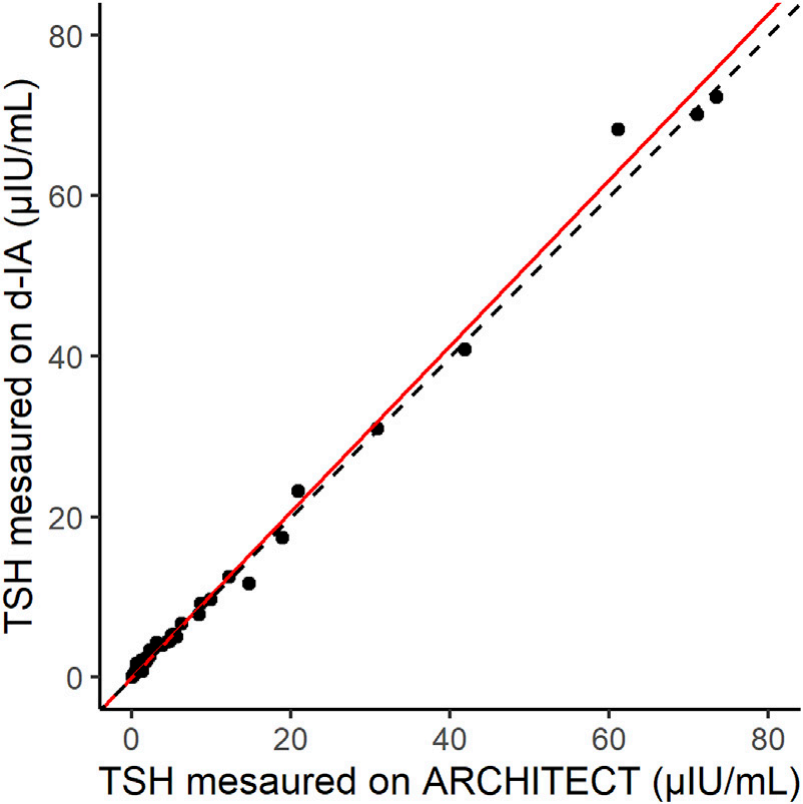
Scatter plot of thyroid-stimulating hormone (TSH) samples measured by ARCHITECT and d-IA. The red line indicates the Passing–Bablok slope. The dashed line indicates the line with a slope of 1 and intercept 0.

The correlation between the results of the ARCHITECT analyzer and d-IA TSH assays was measured in 70 samples, including those from patients with hyperthyroidism (Figure 5). The Passing–Bablok slope was 1.03, and Spearman’s correlation coefficient was 0.97.

### 3.5 Matrix difference

**FIGURE 6:**
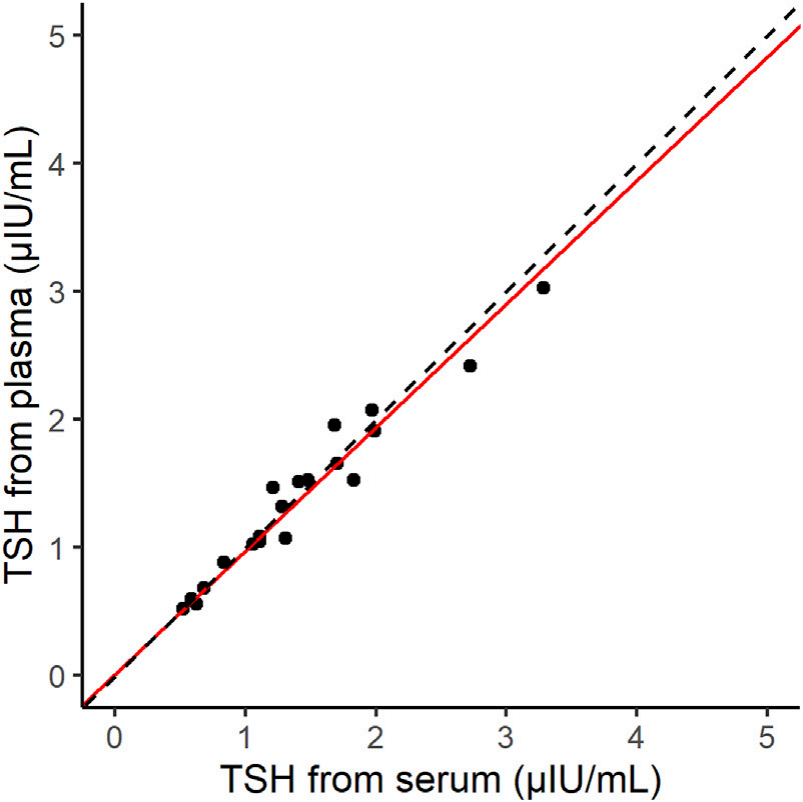
Scatter plot of thyroid-stimulating hormone (TSH) samples derived from serum and plasma on the d-IA platform. The red line indicates the Passing–Bablok slope. The dashed line indicates the line with a slope of 1 and intercept 0.

The correlation between the normal serum and plasma samples (20 samples) was measured by using d-IA (Figure 6). The Passing–Bablok slope was 0.96, and Spearman’s correlation coefficient was 0.97.

## 4 Discussion

In this study, we developed a quantitative TSH assay using a d-IA platform. We used two different monoclonal antibodies, targeting the α- and β-subunits of TSH separately, for the conjugate and beads for detection and capture antibodies, respectively, which enabled two-step immunoreactions. The TSH assay with two-step immunoreaction can reduce crosstalk from other hormones secreted from the pituitary. Taking advantage of the ultrasensitive performance of d-IA (Chiba et al., 2022), we could measure a sample volume of 5 µL. The sample volume needed was similar that required by the currently reported digital ELISA methods (Supplementary Table S1).

Because d-IA counts the number of positive beads derived from pyranine fluorescence, assuming that all beads are positive, i.e., 100% signal, highly concentrated samples cannot be measured. In the case of TSH, concentrations 40 μIU/mL and over could not be distinguished using the signal% (Figure 2). To overcome this problem, the AEB method was utilized (Rissin et al., 2011). The AEB calculates the number of conjugates that form a complex with an analyte. In the original AEB method, fluorescence intensity was used to quantify the number of conjugates. However, in our d-IA platform, although the oil sealing process was applied for all samples at once, the detection of the images was performed sequentially from the first lane to the last one during the enzyme reaction process. Thus, the time difference occurred and that would cause the variation of the fluorescence intensity. Therefore, we used the initial velocity instead of the fluorescence intensity to calculate AEB. Using AEB based on initial velocity, we successfully measured a high concentration of TSH up to 100 µIU/mL, which was similar to the upper range of the current ARCHITECT TSH assay.

Functional sensitivity is the concentration that results in a CV of 20% (Spencer et al., 1996). For the d-IA TSH assay, the LoQ was 0.00228 µIU/mL that was a CV of 20% or less. This functional sensitivity places this assay within the third generation of TSH assays such as ARCHITECT TSH assay (functional sensitivity is 0.0038 µIU/mL). Therefore, our d-IA TSH assay indicated a dynamic range that was the same as that of the ARCHITECT TSH assay. Generally, d-IA technology is considered a next-generation ELISA with ultrahigh sensitivity. However, our d-IA TSH assay did not exhibit ultrahigh sensitivity. One reason for this is that the sample volume of d-IA TSH was only 5 µL. As the amount of TSH is proportional to the sample volume, a low sample volume would include a low amount of TSH. The availability of low sample volumes for digital ELISA has already been established (Supplementary Table S1). Leirs et al., also demonstrated that the low sample volume, digital ELISA, and digital micro fluidics based TSH assay (Leirs et al., 2022). In their study, the sample volume was 1.1 μL and the resultant LoD was 0.0013 μIU/mL that was more sensitive than ARCHITECT assay (LoD is 0.0025 μIU/mL). Secondly, the formation of antigen–antibody complexes depends on the incubation time. (Reverberi and Reverberi, 2007). The 1^st^ and 2^nd^ immunoreaction time for the ARCHTECT TSH assay were 18 min and 4 min, respectively. On the other hand, these times were 3 min and 2 min, respectively, for the d-IA, which increases throughput, but also resulted in low sensitivity. Reducing the number of steps during the assay, such as washing, may contribute to a reduction in the overall assay time, leading to an increase in immunoreaction time. Further improvements in these instruments are required.

In this study, we used pyranine phosphate as the substrate for alkaline phosphatase (Sato et al., 1992). Pyranine is a hydrophilic pH-sensitive fluorescent dye. This hydrophilic property is useful for observation in an oil-sealed microwell chamber because pyranine does not leak into the oil layer (Supplementary Figure S2). The absorption max of pyranine under alkaline conditions is at 460 nm, which is close to the emission wavelength of the blue LED, which can thus be excited efficiently (Avnir and Barenholz, 2005; Pino et al., 2003). The emission peak of pyranine upon blue LED excitation is at 510 nm (Pino et al., 2003). Because color CMOS cameras are highly sensitive to green light, similar to the human eye, the fluorescence of pyranine can be detected efficiently by the camera. In addition, such a large Stokes shift (460–510 nm) enables easy distinction between the excitation and emission wavelengths. Commercially available green fluorogenic substrates, such as fluorescein-diphosphate (FDP), could also be used. However, because the excitation maximum of fluorescein is 490 nm, blue LED cannot excite the dye effectively, leading to low fluorescence intensity and a requirement for high LED power. Unlike FDP, the synthesized pyranine phosphate is catalyzed by alkaline phosphatase in a single reaction, enabling a rapid increase in pyranine in the solution. AttoPhos is another commercially available fluorogenic substrate for alkaline phosphatase. AttoPhos is a monophosphate, like pyranine phosphate, and its peak excitation wavelength is 435 nm, which is slightly too far from the excitation wavelength of the Blue LED. The expected radiant power of Blue LEDs at 435 nm is below 20%, whereas it is close to 100% at 460 nm. Thus, pyranine phosphate was suitable fluorogenic substrate for our d-IA platform.

The correlation between the results of our assay and those of the ARCHITECT TSH assay indicated good agreement (Figure 5). In addition, the difference in the sample matrices indicated a good correlation. Thus, TSH d-IA enabled the measurement of both serum and plasma samples (Figure 6). Although less matrix effect was observed in d-IA in this study, the observed range of TSH was narrow (approximately 1 μIU/mL to 4 μIU/mL), and the sample size was only 20. Therefore, a wider range of TSH levels should be evaluated in further study. The reagent precision was evaluated by measuring four different samples. The precision study data indicated a CV of below 10% in all samples. However, Figure 2 shows a high variation at high TSH sample concentrations. A low sample volume may cause high variation in high-concentration analytes due to the imprecision of pipette aspiration/dispensing and non-specific binding of the samples to tubes and tips (Goebel-Stengel et al., 2011). These could limit the use of low sample volumes, even when using digital ELISA.

In highly concentrated samples, the initial velocity decreased because the fluorescence intensity of multiple conjugates reached a plateau by consuming the substrates immediately. This can lead to unstable parameter estimation. The signal% at 100 µIU/mL decreased slightly. This may be due to failure to detect the positive beads. Indeed, some positive beads were not detected, although they could be seen (Supplementary Figure S3). Since we used the slope of the increase of fluorescence to obtain the positive beads, the values would be low if the slope became shallow. To avoid this, the enzyme reaction process should be observed immediately after sealing the microwells with oil or by increasing the concentration of the fluorogenic substrate. However, if the concentration of the analyte were to be increased, a shorter observation period would be required. These are the fundamental limitations of our current d-IA method.

We also evaluated the ratio of bright wells without beads for precise signal detection. The bright wells without beads included combinations of 1) true-negatives (TN) of beads and true-positives (TP) of bright wells, 2) false-negatives (FN) of beads and TP of bright wells, 3) TN of beads and false positives (FP) of bright wells, and FN of beads and FP of bright wells. To evaluate the number of cases, we calculated the ratio of bright wells without beads to empty wells, $\left(F-M\right)/\left(W-B\right)$ , where *F* is the total number of bright wells, *M* is the number of bright wells with beads, *W* is the total number of microwells in the analysis area, and *B* is the number of beads (Supplementary Figure S4A). *W* was calculated by the number of microwells per μm^2^ multiplied by the analysis area. Under our experimental conditions, *W* = 117,385 microwells (calculated by the diameter of analysis area, 3,237.9 μm, and the number of microwell in unit area was approximately 0.001426 microwell/μm^2^). Supplementary Figure S4A indicates that the ratio increased depending on the TSH concentration. At 0 μIU/mL of TSH, the mean rate was 0.003%, suggesting the efficiency of washing to eliminate conjugate contamination. We also calculated the dropping rate of bright wells as $\left(F-M\right)/F$ (Supplementary Figure S4B). The ratio indicated a rate of 0.1–0.2 in the presence of TSH samples, and no TSH concentration tendency was observed. Such dropping would occur in the above four cases (Supplementary Figure S4C), and we speculated on the reason for this. FN of beads is caused by the missed detection of beads. Although we detected the beads based on green LED scatter, the intensity of beads varied due to the fluctuation of the beads inside of the microwell and aberration of the optical system. Because our algorithm uses a global threshold to detect the beads, low-intensity beads were missed. To avoid this, further improvements in the optical system (more uniform scattering light illumination and low-aberration optics) and detection algorithms are required. FP of bright wells was due to debris or pooling of the substrate on the microwells, TP of bright wells may be due to conjugate contamination in the final substrate buffer. Because the dropping rate was constant relative to the TSH concentration (except at 0 μIU/mL), the rate of bright wells without beads indicated the dependence on the TSH concentration.

In this assay, the immunoreaction times for the first and second reactions were 3 and 2 min, respectively. However, the total assay time was approximately 40 min. The time-consuming steps are the B/F separations. Optimization of the assay protocol is desirable to reduce the assay time.

A high-sensitive fourth generation TSH assay, which has a functional sensitivity of 0.001 μIU/mL, could predict the results of the TSH fold-response upon TRH stimulation, which is used to evaluate subclinical hyperthyroidism, leading to elimination of the TRH stimulation test, which causes side effects, such as nausea (Spencer et al., 1993). We believe that the d-IA TSH assay has the potential to extend beyond the fourth generation of TSH assay kits. However, functional sensitivity is a tradeoff between sample volume and immunoreaction time. Further improvement in d-IA TSH is desirable to satisfy the requirements of sample volume, immunoreaction time, and functional sensitivity.

Our TSH d-IA assay used a sample volume of only 5 µL. Therefore, if a small sample volume is obtained such as using a fingerstick (approximately 20 µL whole blood), it would lead to reduce the burden on the patient. Point-of-care technology and telemedicine applications could be achieved by combining our assay system with other devices.

## Data Availability

The datasets presented in this article are not readily available because the data is confidential. Requests to access the dataset should be directed to the corresponding author.

## Abbreviations

AEB, average number of enzymes per bead; ASD, assay specimen diluent; B/F, bind/free; CV, coefficient of variation; d-IA, digital immunoassay; ELISA, enzyme-linked immunosorbent assay; FN, false negative; FP, false positive; LoB, limit of blank; LoD, limit of detection; LoQ, limit of quantification; SD, standard deviation; T3, triiodothyronine; T4, thyroxin; TN, true negative; TP, true positive; TRH, thyrotropin-releasing hormone; TSH, thyroid stimulating hormone.
