# Supplementary material for: Development of a quantitative thyroid-stimulating hormone assay system for a benchtop digital ELISA desktop analyzer
Source: Front Bioeng Biotechnol. 2023 Sep 21;11:1227357. doi: 10.3389/fbioe.2023.1227357 (PMC10551129; doi:10.3389/fbioe.2023.1227357)
Supplement: Supplementary file 1 [file DataSheet1.pdf]

Supplementary Material

Development of a quantitative thyroid-stimulating hormone assay system for a benchtop digital ELISA desktop analyzer

Yoshiyuki Arai†, Dong Wang†, Miki Takeuchi†, Sosuke Utsunomiya†, Takuma Degawa, Atsushi Kai, Hisashi Ichikawa, Ryotaro Chiba, Toru Yoshimura*

†These authors contributed equally to this work and share the first authorship

*** Correspondence:** Toru Yoshimura: tohru.yoshimura@abbott.com

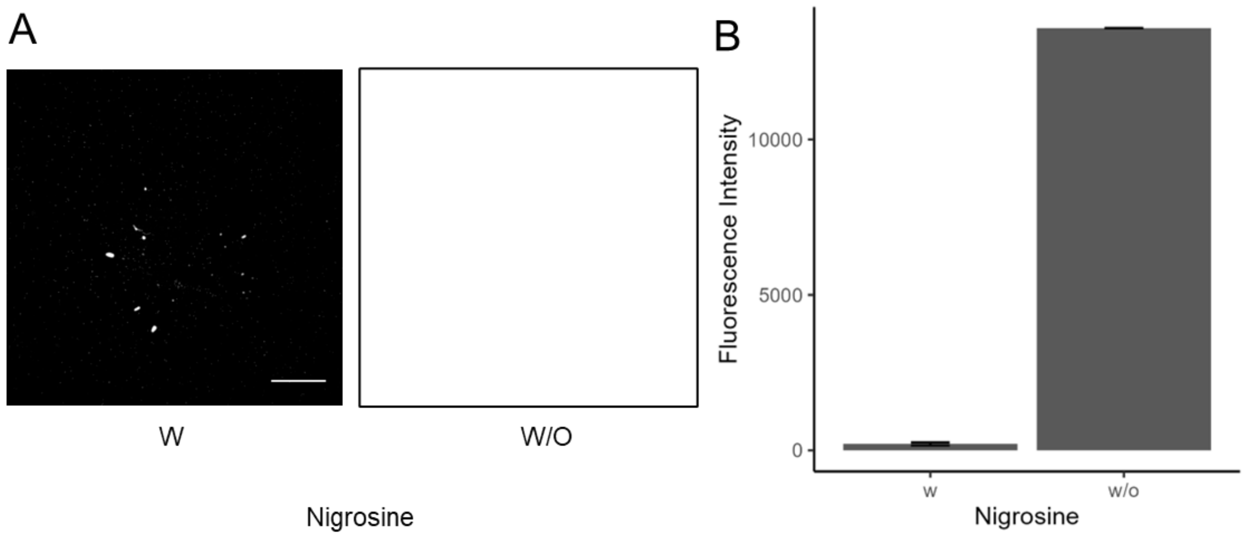

Supplementary Figure 1. (A) Comparison of the fluorescence intensities with (W) and without (W/O) nigrosine. (**B**) Typical fluorescence images of d-IA with and without nigrosine. TSH: 0.5 µIU/mL. Comparison of the fluorescence intensity of the entire image area, n = 4. Error bars represent standard deviation.

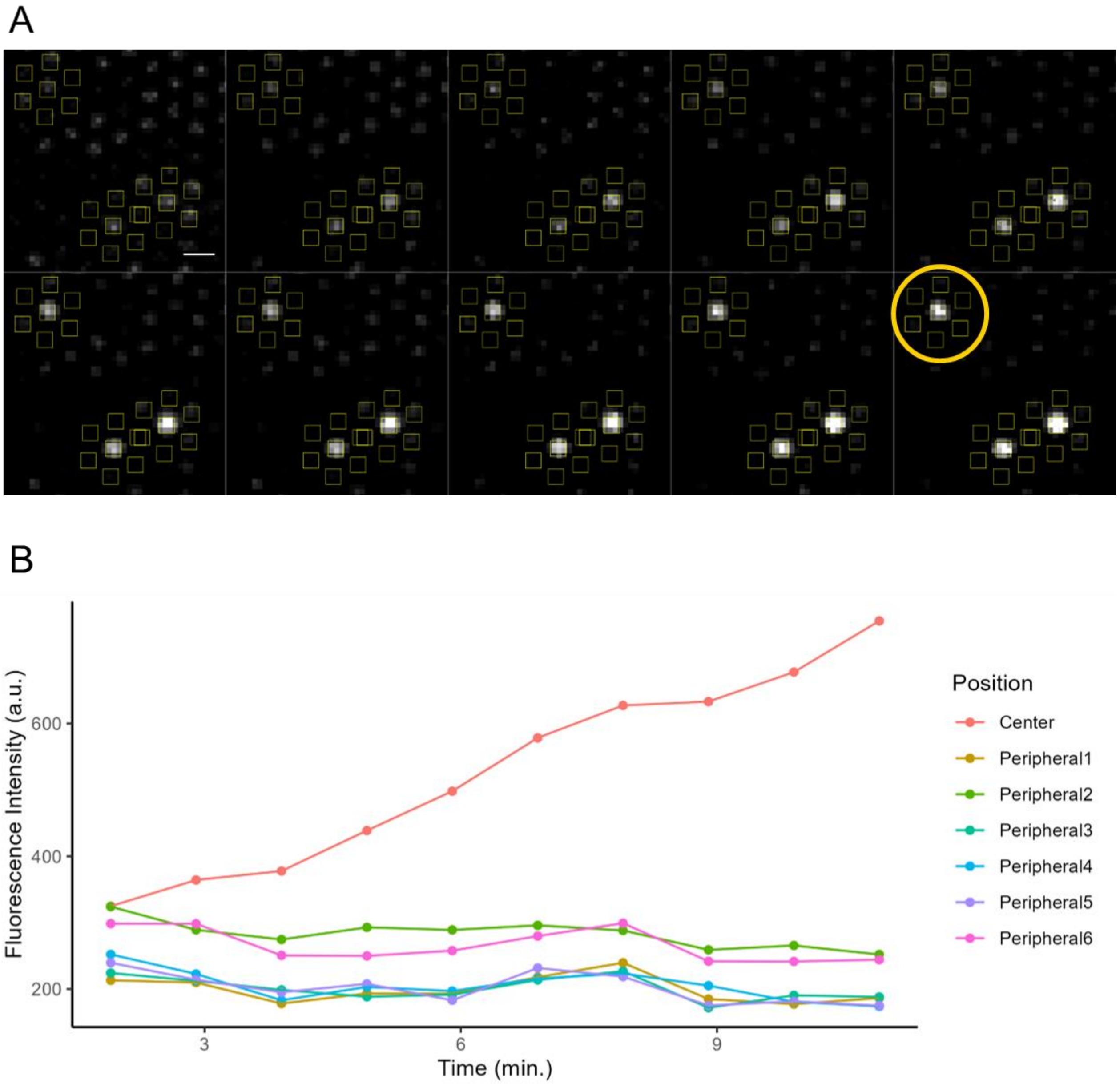

Supplementary Figure 2. Cross-contamination of pyranine fluorescence after oil sealing. (**A**) Montage of fluorescent images. Yellow squares indicated the regions of interest (ROIs) of the microwells where isolated positive particles were located. Scale bar = 10 μm. (**B**) Time trajectories of fluorescence intensity after oil sealing the microwells shown in (**A**) as a yellow circle. The mean fluorescence intensity at each ROI was extracted using the ImageJ software.

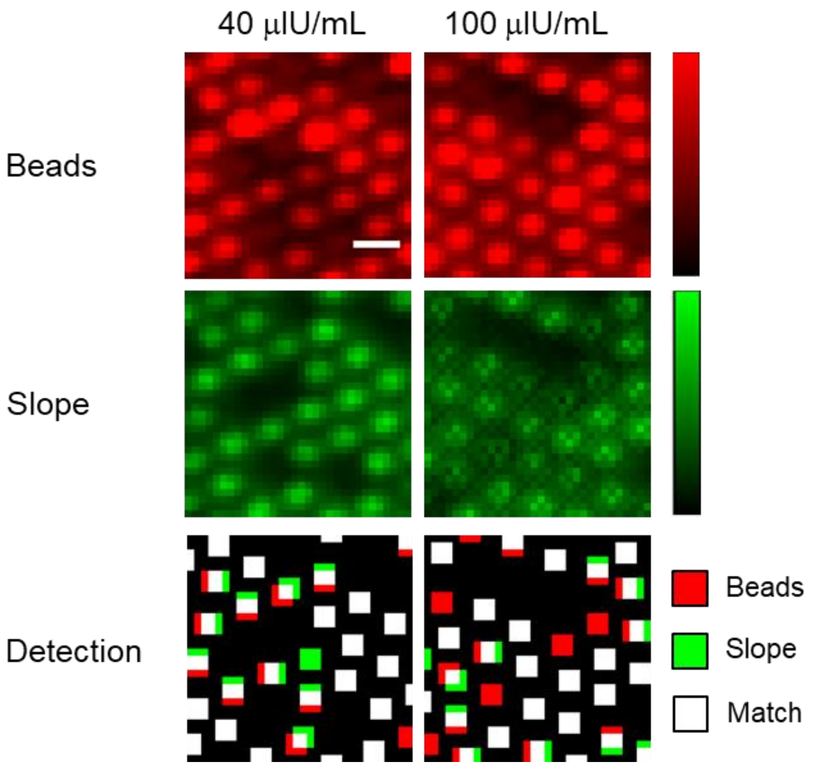

Supplementary Figure 3. Images of beads, slope, and resultant detected data from 40 μIU/mL and 100 μIU/mL TSH samples. The detected images are the outputs of the image analysis. The centers of the beads and slope particles were detected as described in the Methods section. White squares indicate the overlap of the detected beads and slopes. Scale bar, 10 μm.

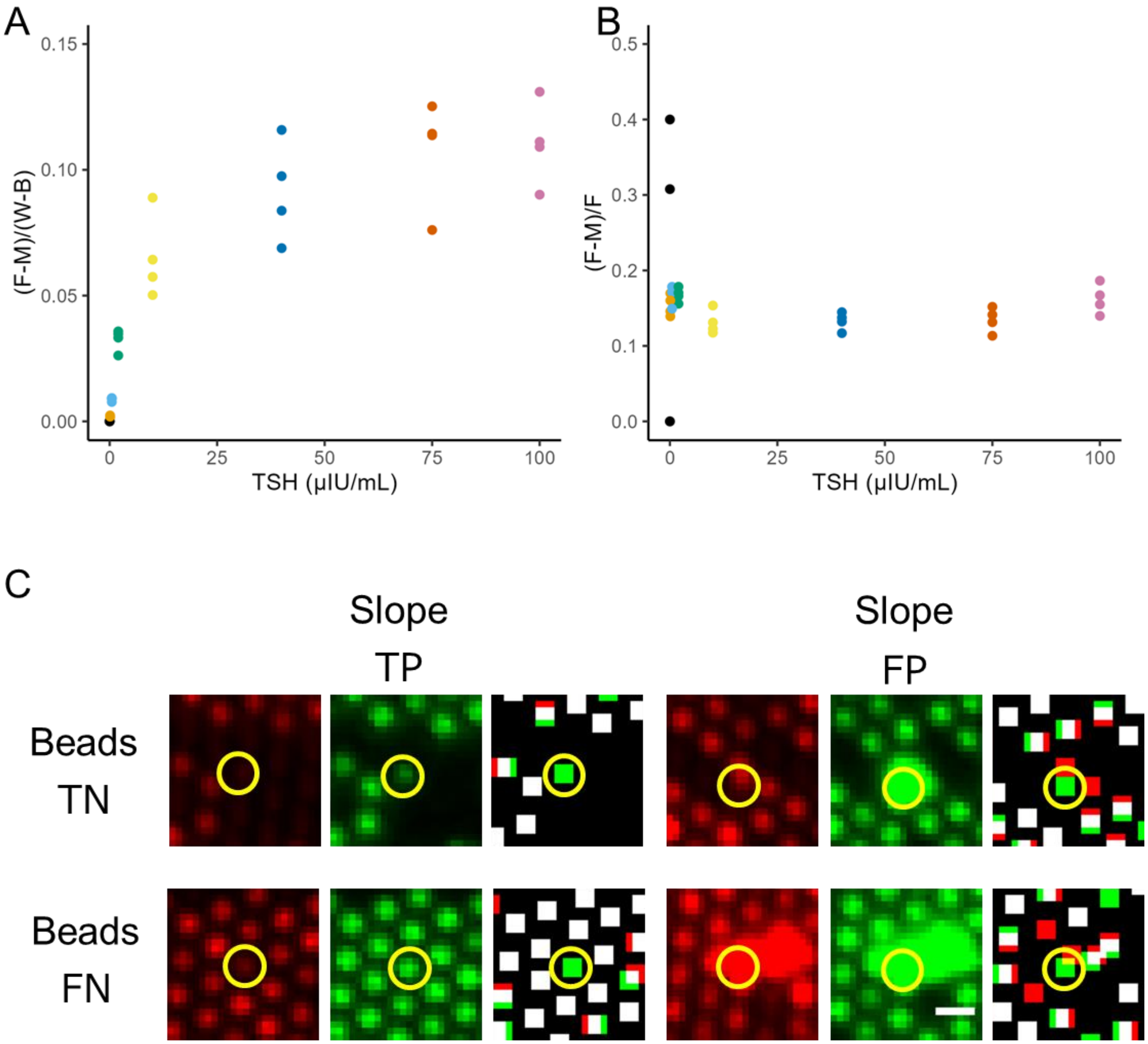

Supplementary Figure 4. Ratio of bright wells without beads and images. (**A**) Ratio of bright wells without beads to empty wells. F: Total number of bright wells, M: Number of bright wells with beads, W: Total number of microwells, B: Number of beads. (**B**) Ratio of bright wells without beads to total number of bright wells. (**C)** Example images of beads, bright wells (Slope), and the result at each combination. TN: True Negative, FN: False Negative, TP: True Positive, FP: False Positive. Yellow circles indicated the bright wells without bead in each combination. Scale bar, 10 μm.

Supplementary Table 1. Comparison of sample volume among digital ELISA technologies

Technology	Biomarker	Sample volume	Reference
**SiMoA ***	**PSA**	**5-100 μL ****	**(Rissin et al., 2010)**
**Droplet digital ELISA**	**IFNγ**	**100 μL**	**(Cohen et al., 2020)**
**Droplet-free digital ELISA**	**HBsAg**	**75 μL**	**(Akama et al., 2016)**
**PEdELISA**	**10 Multiplex**	**10 μL**	**(Song et al., 2021)**
**Digital ELISA on DMF**	**TSH**	**1.1 μL**	**(Leirs et al., 2022)**
**d-IA (this paper)**	**TSH**	**5 μL**	

*** SiMoA is commercially available from Quanterix.**

**** Volumes shown are for at bench dilutions. On-instrument dilutions require higher sample volumes (described in https://www.quanterix.com/wp-content/uploads/2022/04/21.07.20_Quanterix_HD_1_HD_X_assay_menu.pdf)**

References

Akama K, Shirai K, Suzuki S. Droplet-Free Digital Enzyme-Linked Immunosorbent Assay Based on a Tyramide Signal Amplification System. Anal Chem. 2016;88(14):7123-9. doi: 10.1021/acs.analchem.6b01148

Cohen L, Cui N, Cai Y, Garden PM, Li X, Weitz DA, et al. Single Molecule Protein Detection with Attomolar Sensitivity Using Droplet Digital Enzyme-Linked Immunosorbent Assay. ACS Nano. 2020;14(8):9491-501. doi: 10.1021/acsnano.0c02378

Leirs K, Dal Dosso F, Perez-Ruiz E, Decrop D, Cops R, Huff J, et al. Bridging the Gap between Digital Assays and Point-of-Care Testing: Automated, Low Cost, and Ultrasensitive Detection of Thyroid Stimulating Hormone. Anal Chem. 2022;94(25):8919-27. doi: 10.1021/acs.analchem.2c00480

Rissin DM, Kan CW, Campbell TG, Howes SC, Fournier DR, Song L, et al. Single-molecule enzyme-linked immunosorbent assay detects serum proteins at subfemtomolar concentrations. Nat Biotechnol. 2010;28(6):595-9. doi: 10.1038/nbt.1641

Song Y, Sandford E, Tian Y, Yin Q, Kozminski AG, Su SH, et al. Rapid single-molecule digital detection of protein biomarkers for continuous monitoring of systemic immune disorders. Blood. 2021;137(12):1591-602. doi: 10.1182/blood.2019004399
